# Benthic protists and fungi of Mediterranean deep hypsersaline anoxic basin redoxcline sediments

**DOI:** 10.3389/fmicb.2014.00605

**Published:** 2014-11-12

**Authors:** Joan M. Bernhard, Konstantinos Kormas, Maria G. Pachiadaki, Emma Rocke, David J. Beaudoin, Colin Morrison, Pieter T. Visscher, Alec Cobban, Victoria R. Starczak, Virginia P. Edgcomb

**Affiliations:** ^1^Geology and Geophysics Department, Woods Hole Oceanographic InstitutionWoods Hole, MA, USA; ^2^Department of Ichthyology and Aquatic Environment, School of Agricultural Sciences, University of ThessalyVolos, Greece; ^3^Division of Life Sciences, Hong Kong University of Science and Technology, Clear Water BayKowloon, Hong Kong; ^4^Biology Department, Woods Hole Oceanographic InstitutionWoods Hole, MA, USA; ^5^Biology Department, University of Nevada RenoReno, NV, USA; ^6^Department of Marine Sciences, University of ConnecticutGroton, CT, USA

**Keywords:** eukaryote, DHABs, discovery, Urania, L’ Atalante, diversity, rRNA

## Abstract

Some of the most extreme marine habitats known are the Mediterranean deep hypersaline anoxic basins (DHABs; water depth ∼3500 m). Brines of DHABs are nearly saturated with salt, leading many to suspect they are uninhabitable for eukaryotes. While diverse bacterial and protistan communities are reported from some DHAB water-column haloclines and brines, the existence and activity of benthic DHAB protists have rarely been explored. Here, we report findings regarding protists and fungi recovered from sediments of three DHAB (Discovery, Urania, L’ Atalante) haloclines, and compare these to communities from sediments underlying normoxic waters of typical Mediterranean salinity. Halocline sediments, where the redoxcline impinges the seafloor, were studied from all three DHABs. Microscopic cell counts suggested that halocline sediments supported denser protist populations than those in adjacent control sediments. Pyrosequencing analysis based on ribosomal RNA detected eukaryotic ribotypes in the halocline sediments from each of the three DHABs, most of which were fungi. Sequences affiliated with Ustilaginomycotina Basidiomycota were the most abundant eukaryotic signatures detected. Benthic communities in these DHABs appeared to differ, as expected, due to differing brine chemistries. Microscopy indicated that only a low proportion of protists appeared to bear associated putative symbionts. In a considerable number of cases, when prokaryotes were associated with a protist, DAPI staining did not reveal presence of any nuclei, suggesting that at least some protists were carcasses inhabited by prokaryotic scavengers.

## INTRODUCTION

Redox boundaries in marine sediments can have significant geochemical gradients, transitioning from fully aerated to anoxic (lack of detectable dissolved oxygen) conditions within short vertical distances (e.g., Cai and Sayles, 1996; Yucel, 2013). These chemoclines are zones of intense biogeochemical cycling, involving all major elements including carbon, oxygen, nitrogen, sulfur, and hydrogen as well as iron and manganese. While considerable effort has been dedicated to studying the biogeochemistry of redox boundaries of marine sediments in neritic zones (e.g., Yucel, 2013), silled basins (e.g., Reimers et al., 1996; Bernhard et al., 2003), and hydrocarbon seeps (e.g., Orcutt et al., 2005), less is known regarding chemoclines in the deep bathyal to hadal zones.

Microbial eukaryotes inhabiting marine chemoclines can be numerous compared to those from nearby more aerated sites (e.g., Bernhard et al., 2000; Edgcomb et al., 2011c). Additionally, marine chemocline microbial eukaryotes typically have associated prokaryotes existing as endobionts and/or ectobionts (e.g., Esteban et al., 1995; Fenchel and Finlay, 1995; Bernhard et al., 2000; Bernhard, 2003). Generally, in the cases receiving dedicated study, evidence suggests these associations are mutualistic or commensal. Investigations into the systematics and physiologies of the partners in these putative symbioses often yield surprising results, with multiple structured associations (Camerlenghi, 1990; Edgcomb et al., 2011c) and novel cellular adaptations (e.g., Bernhard and Bowser, 2008; Bernhard et al., 2010a).

**FIGURE 1:**
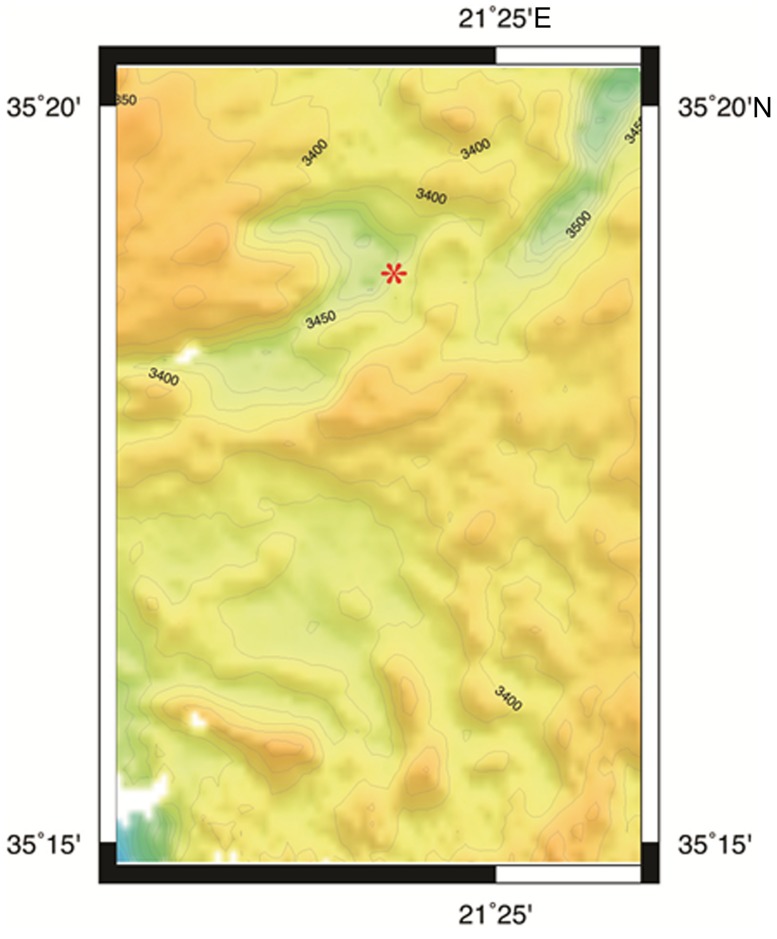
**Bathymetric map of L’ Atalante DHAB generated by Seabeam.** Red asterisk = approximate sample site.

The deep bathyal Mediterranean has numerous deep hypersaline anoxic basins (DHABs; **Figure 1** and also see Figure 1 in Stock et al., 2013b), which are brine-filled bathymetric depressions formed from the dissolution of subterranean Miocene salt deposits exposed to seawater after tectonic activity (Camerlenghi, 1990). Due to the sequence of different chemical ions precipitating from seawater as it evaporates, different layers of those salt deposits are characterized by differing chemistries. It follows that DHABs differ in brine chemistry. In the three DHABs studied here, Urania brine is the highest in free sulfide and methane, Discovery brine is highest in chlorine and magnesium, and L’ Atalante brine is highest in sodium, potassium, and sulfate (**Table 1**). Further details on the brine chemistries are available in van der Wielen et al. (2005).

**Table 1:** Geochemical data of DHAB brines and typical seawater (modified from van der Wielen et al., 2005).

Geochemistry	Urania	Discovery	L’ Atalante	Seawater
Coordinates (N) (E)	35 13.784; 21 28.943	35 17.150; 21 42.308	35 18.865; 21 24.338	-
Water depth (m)	3468	3582	3430	-
Density (10^3^ kg m^-3^)	1.13	1.35	1.23	1.03
Na^+^ (mM)	3503	68	4674	528
Cl^-^ (mM)	3729	9491	5289	616
Mg^2+^ (mM)	316	4995	410	60
K^+^ (mM)	122	20	369	11
SO_4_^2-^ (mM)	107	96	397	32
HS^-^ (mM)	16	1	2.9	2.6 × 10^-6^
CH_4_ (mM)	5.6	0.03	0.5	1.4 × 10^-6^

*Coordinates and water depths reflect our approximate sampling area for each DHAB*.

**FIGURE 2:**
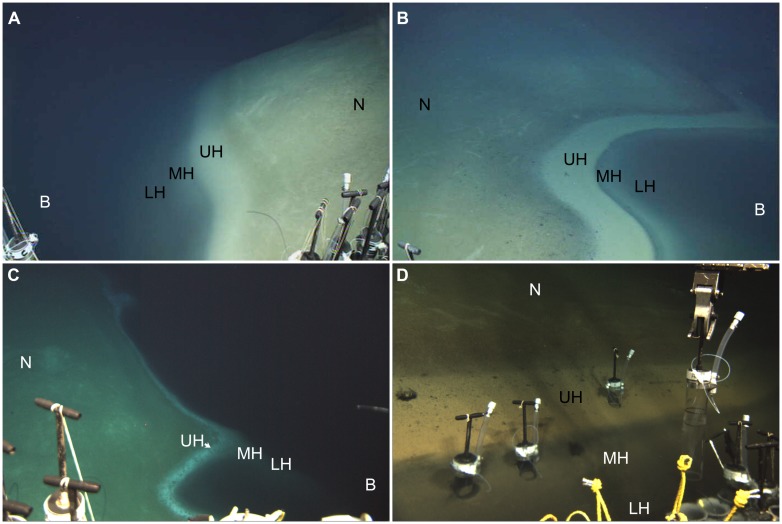
**Underwater photographs showing small portion of redoxcline impinging the seafloor in Urania **(A)**, Discovery **(B,D)**, and L’ Atalante **(C)** DHABs, eastern Mediterranean.** Note red laser spots on seafloor in **(C)**; dots are spaced 10 cm apart. N = normoxic control region; UH = upper halocline zone; MH = mid-halocline zone; LH = lower halocline zone; B = brine. Injector cores emplaced in Discovery upper and mid-halocline **(D)** illustrate the ability to sample different zones. All images © Woods Hole Oceanographic Institution.

These DHABs have been the topic of attention over the last decade. In particular, the water columns overlying these bathymetric features have received considerable study, for both prokaryote communities (van der Wielen and Heijs, 2007; Ferrer et al., 2012) and eukaryote communities (e.g., Alexander et al., 2009; Edgcomb et al., 2009, 2011d; Stock et al., 2013a). The sediments overlain by the brines have also been investigated for their prokaryotic (e.g., van der Wielen and Heijs, 2007; Yakimov et al., 2007; Ferrer et al., 2012) as well as metazoan constituents (Danovaro et al., 2010). The benthic redoxclines of DHABs have received less study (e.g., the study of Red Sea brine pool benthic eukaryotes by Wang et al., 2014), probably due to their limited spatial extent. Haloclines of DHABs are typically ∼2 m in vertical thickness (van der Wielen et al., 2005; Hallsworth et al., 2007; Yakimov et al., 2007). Thus, when the halocline impinges the seafloor, the pitch of the bathymetric slope directly impacts the lateral manifestation of the halocline (redoxcline) on the seafloor (**Figure 2**).

The goal of this study was to investigate the microbial eukaryotes in the redoxcline of Mediterranean DHAB sediments. Here we report results describing the eukaryotic community composition and abundances. We include results from classic morphologic analyses as well as from RNA-based sequencing efforts and a fluorescence-labeling approach to identify active eukaryotes. For comparison, we include data on microbial eukaryote communities obtained from adjacent normoxic sediments.

## MATERIALS AND METHODS

### SAMPLE COLLECTION AND SALINITY

Samples were collected using the ROV *Jason* from 24 November to 6 December 2011. Most sediments for this study were collected with the typical Deep Submergence Lab (http://www.whoi.edu/groups/DSL/) *Alvin*-type pushcores (6.35-cm diameter; hereafter referred to as “cores”) configured with a seal to prevent contamination during ascent. Cores were collected from nearby (within 100 m) aerated sediments, just upslope from each DHAB. Thus, control samples at Urania and Discovery were consistently from 1 to 3 m shallower water depths than the halocline samples. Because ROV *Jason* is neutrally buoyant, it was not possible to core deep in the DHAB brine. Thus, cores of deep halocline sediments were obtained by *Jason* reaching toward the brine with the ROV manipulator. The depth differential between halocline samples did not exceed 2 m. Given the waters depths for all samples were >3400 m, these depth differences are negligible.

Upon ROV *Jason* recovery, all cores were taken as quickly as possible (within 5 min) to the RV *Atlantis* environmental room set at 9.5°C (±0.5°C), until further processing. Cores for microelectrode profiling were placed in an N_2_-flushed glove bag and profiled as described below. Cores for quantification (counts) and imaging were first sampled for their bottom-water salinity, measured via hand-held refractometer. Many samples needed to be diluted up to 10-fold to register on the refractometer. The hand-held refractometer was not expected to provide precise salinity data, especially for the very high MgCl_2_ brine of Discovery, but was intended as a relative indicator between samples from different halocline horizons in any given DHAB. After salinity was recorded, each of these cores was sectioned into 1-cm intervals to a depth of 3 cm. Each sediment slice was preserved in either 3.8% formaldehyde or 3% TEM-grade glutaraldehyde in a 0.1 cacodylic acid sodium salt buffer. In some cases, there was no overlying bottom water due to full penetration or over-penetration of the corer. In these cases, salinity data are not reported. In instances where no salinity data was obtained from any given core, we estimated salinity using published literature (**Table 1**) and confirmed by our own water column high-range CTD data (collected for a separate water-column study, data not shown), extrapolated for specific core location relative to others with known salinity and the halocline boundary. We designate halocline samples by their relative location (depth, refractometer data) in the following descending order: upper halocline, mid-halocline, lower halocline.

To avoid the possibility that pressure and temperature changes during ascent may cause death of some fraction of the eukaryotic community, a subset of cores were incubated *in situ* using the viability indicator CellTracker^TM^ Green CMFDA (chlorofluoromethyl fluorescein diacetate; Life Technologies). CellTracker Green relies on active esterases, which are known to function in at least one DHAB brine (Ferrer et al., 2005). For these samples, push corers were emplaced on the seafloor and left for ≥24 h prior to collection. These pushcores were modified to allow injection of the CellTracker Green using the ROV manipulator. Similar “injector” cores have been employed before for seafloor incubations using the same fluorescent probe (Bernhard et al., 2009). During the next *Jason* dive (2–3 days later), these cores were removed from the seafloor per standard procedure and brought to the sea surface. Because only one dive was executed in L’ Atalante, those incubations were begun early on the dive and terminated after recovery later that day, resulting in incubation times of approximately 12.5 h. The injector cores were sectioned and preserved in glutaraldehyde as noted above.

### SEDIMENT PROFILING AND SAMPLING FOR RNA EXTRACTION

In the environmental room on RV *Atlantis*, designated cores containing overlying site water were profiled for dissolved oxygen with a vertical resolution of 0.25 mm in a N_2_-filled glove bag using polarographic microelectrodes (Visscher et al., 1991). These cores were then subsampled from each core center using a sterile 20-ml syringe (∼1.4 cm inside diameter) with the Luer end removed. These syringe cores were frozen at -80°C and transported to WHOI, where they were slightly thawed to enable slicing of the subcore. The surface 2 cm of the subcore was placed in conical centrifuge tubes and stored at -80°C until RNA extraction.

### CHARACTERIZATION OF SEDIMENT EUKARYOTES USING SMALL SUBUNIT RIBOSOMAL RNA (SSU rRNA)

RNA from approximately 8 g of the top 2 cm from each frozen syringe subcore was extracted using an optimized protocol with the RNA Power Soil kit (MoBio, USA). Major modifications included introduction of three cycles of freeze-thaw (-80°C, 5 min., 65°C, 5 min.), bead beating with 2 × 5 min. intervals on a horizontal vortexer, an overnight nucleic acid precipitation, and a 1-hour centrifugation during the precipitation step. In addition, we introduced two DNAase treatments using TurboDNAase (Ambion, USA). Removal of DNA was confirmed by PCR using general eukaryotic primers. RNA was purified using the MEGAclear kit (Ambion, USA). Reverse transcription of the purified RNA samples was performed using the QuantiTect kit (Qiagen, USA). Tag-pyrosequencing of the eukaryotic small subunit rRNA (18S rRNA) gene was performed using PCR amplification of the V4 region of the 18S rRNA gene and the primer pair TAReuk454FWD1 (5′-CCAGCA(G/C)C(C/T)GCGGTAATTCC-3′) and TAReukREV3 (5′-ACTTTCGTTCTTGAT(C/T)(A/G)A-3′). In brief, a one-step, 30-cycle PCR reaction was performed using GoTaq polymerase (Promega, USA). PCR conditions included: 94°C for 3 min, followed by 30 cycles of 94°C for 30 s; 55°C for 40 s; 72°C for 1 min; and a final elongation step at 72°C for 5 min. Following PCR, all amplicon products (ca. 450 bp) from different samples were purified using the MinElute Reaction Cleanup kit (Qiagen, USA). In parallel, blank extractions and amplification reactions were performed to check for contamination. Distinct tags (multiplex identifiers) were used for each of the samples. Amplicon libraries were sequenced utilizing the Roche 454 FLX Titanium platform and reagents following manufacturer’s guidelines at the MR DNA (Molecular Research LP, Shallowater, TX, USA) sequencing facility.

Denoising of the flowgrams was performed using Acacia (Bragg et al., 2012). Processing of the resulting sequences, i.e., trimming and quality control was performed using QIIME (Caporaso et al., 2010). Sequences with ≥380 bp and no ambiguous base calls and no homopolymers ≥6 bp were included in further analysis. All sequences were binned into operational taxonomic units (OTUs) and were clustered (average neighbor algorithm) at 97% sequence similarity identity. Taxonomic assignments were made using BLAST within QIIME. Sequences have been deposited in the GenBank SRA archive under the accession number SRP049010.

### QUANTIFICATION AND IMAGING OF PROTISTS USING MICROSCOPY

At WHOI, pushcore samples preserved in either glutaraldehyde or formalin on the ship were used for morphologic documentation and/or quantification. Percoll density gradients were used to isolate the eukaryotes (Starink et al., 1994), subjecting to each established gradient 0.5 ml of sample from the surface 1 cm. The eukaryote-laden supernatant of each density gradient was filtered onto one 2-μm blackened polycarbonate filters and mounted with Citifluor AF1: Vectashield: 1× PBS (Phosphate buffered saline; 11:2:1) media containing 1 mg/ml DAPI (4′,6-diamidino-2-phenylindole) on a microscope slide under a coverslip. Eukaryotes were counted from each filter [up to 60 fields were counted at 400× magnification on a Zeiss Axio Imager M2 upright microscope with differential interference contrast (DIC) and phase contrast optics, until 350 cells per filter were counted]. For each sample, eukaryotes were isolated from three separate Percoll gradients. Thus, three replicate filters were counted for each sample.

Because the filter impedes imaging, the specimen-laden side of a subset of filters from selected samples was blotted onto a drop of mounting media atop a microscope slide, covered with a coverslip and then sealed. The mounted slide was scanned for eukaryotes appropriate for imaging (clean, larger, mostly unobstructed specimens). Images were taken either with an Olympus BX51 upright epifluorescence microscope with DIC and phase contrast optics and an Olympus DP70 color digital camera or the Zeiss Axio Imager M2 and a Canon EOS Rebel color digital camera.

Non-quantitative aliquots of samples incubated on the seafloor with CellTracker Green were similarly subjected to Percoll extraction, DAPI staining and/or imaging, although specimens were also examined under 480-nm excitation and 520-nm emission to determine if they had active esterases at the time of incubation.

## RESULTS

### PORE-WATER DISSOLVED OXYGEN

**FIGURE 3:**
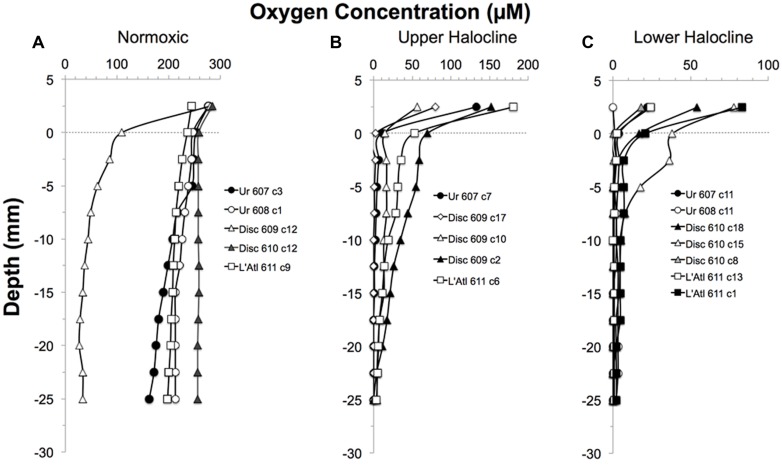
**Depth profiles of the oxygen concentrations in overlying waters and sediments of pushcores collected for this study, presented by DHAB and general habitat.** From left to right: Normoxic (control;**A**); Upper Halocline**(B)**; Lower Halocline**(C)**.

Dissolved oxygen in control cores remained detectable to depths deeper than 1 cm (**Figure 3A**). In all but one of the halocline cores, oxygen was detectable in overlying waters (**Figures 3B,C**). At the sediment-water interface of halocline cores, [O_2_] was typically below 21 μM. Oxygen remained detectable to at least 2.0 cm depth in 7 of the 12 halocline cores. In some halocline cores (i.e., L’ Atalante dive 611 core 1 and core 6; Discovery dive 609 core 10), oxygen remained detectable at the deepest measured horizon (2.5 cm). Oxygen was near the detection limit (<3 μM) throughout the top 2.5 cm in core 608 c 11 (Urania); this core was not analyzed for eukaryotes.

### BOTTOM-WATER SALINITY

Salinity in bottom waters within 10 cm of the sediment–water interface of cores from Discovery and L’ Atalante varied widely. Salinity was not measured from cores collected in and near Urania DHAB. Control cores had bottom-water salinities of 41–50. The highest salinity measured was 220 (L’ Atalante; **Table 2**); the highest salinity measured in Discovery was 146. We reiterate that these salinities do not represent brine characteristics *per se* but allow relative comparisons between pushcores collected within each halocline.

**Table 2:** Samples from cruise AT18-14 analyzed for counts, imaging (DAPI and/or CTG imaging), and rRNA sequencing.

DHAB	Habitat	Salinity	Sample	(dive#, core#)	Type of core	Collection date, 2011	Interval analyzed (cm)	Analysis
Urania	Normoxic control	ND	607-08, cB	Injector pushcore	30 November	0–1	Counts
	Normoxic control	ND	608, cl	Pushcore	30 November	0–2	rRNA
	Halocline	ND	607-08, cE	Injector pushcore	30 November	0–1	Counts
	Halocline	ND	608, c09	Pushcore	30 November	0–2	rRNA
Discovery	Normoxic control	ND	609, cl2	Pushcore	2 December	0–2	rRNA
	Mid-halocline	ND	609, cl7	Pushcore	2 December	0–2	rRNA
	Mid-halocline	85	610, cl4	Pushcore	3 December	0–1	Counts
	Mid-halocline	102	609-610, cH	Injector pushcore	3 December	0–1	Imaging
	Lower halocline	ND	610, c9	Pushcore	3 December	0–1	Imaging
	Lower halocline	100	610, cl5	Pushcore	3 December	0–2	rRNA
	Lower halocline	106	609-10, cL	Injector pushcore	3 December	0–1	Counts
L’ Atalante	Upper halocline	41	611, c06	Pushcore	6 December	0–2	rRNA
	Upper halocline	44	611, c5	Pushcore	6 December	0–1	Imaging
	Mid-halocline	53	611, clO	Pushcore	6 December	0–1	Counts
	Lower halocline	100	611 cl3	Pushcore	6 December	0–2	rRNA
	Lower halocline	220	611, cl7	Pushcore	6 December	0–1	Counts

*All cores analyzed for rRNA were also profiled for oxygen. Additional cores that were profiled for oxygen but not used for any faunal analyses are omitted. Salinity data are based on refractometer readings, which are likely inaccurate for athalassohaline brines such as Discovery. ND = no data*.

### PROTIST MORPHOTYPES

**FIGURE 4:**
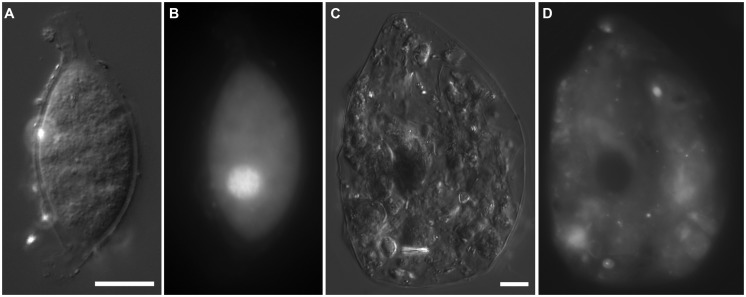
**Light micrographs. (A,B)** Thecate foraminifer from 611 c3 (L’ Atalante control), paired images showing DIC **(A)** and epifluorescence **(B)** of DAPI staining. Note the nucleus and lack of associated prokaryotes **(B)** in the well-vacuolated cytoplasm **(A)**. This specimen was considered living at the time of fixation. **(C,D)** protist carcass from 609-610 cL (Discovery lower halocline), paired images showing DIC **(C)** and epifluorescence **(D)** of DAPI staining. Note the lack of a DAPI labeled nucleus and presence of a few prokaryotes. This specimen was considered dead at the time of fixation. Scales = 10 μm.

**FIGURE 5:**
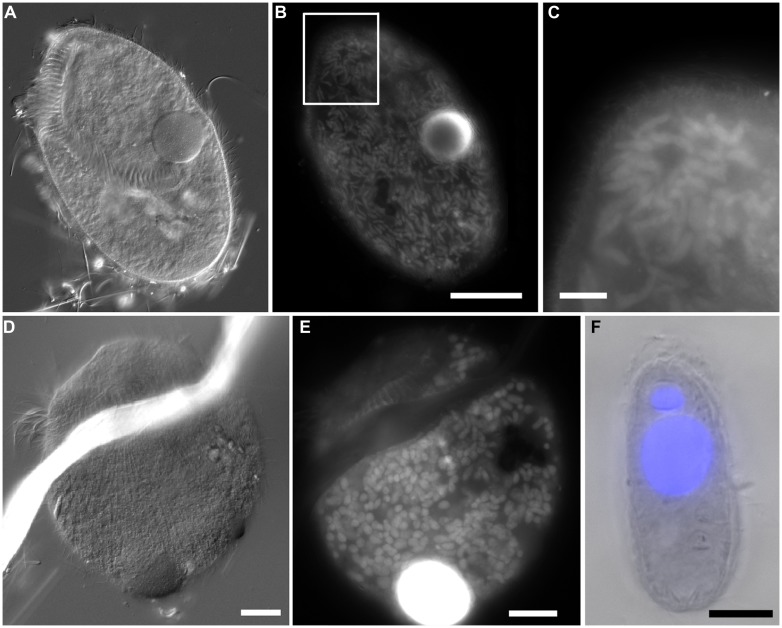
**Light micrographs of ciliates from Urania halocline. (A,C)** Possible armophorean, *Metopus*-like ciliate from 608 c11. **(A,B)** Paired images showing DIC **(A)** and epifluorescence **(B)** of DAPI staining. Box in **(B)** shows area depicted in **(C)**. Note the nucleus **(B)** and endobionts **(B,C)**. **(D,E)** Possible karyorelictid or hypotrich/strichothrich ciliate from 607 c10. DIC image **(D)** of large endobiont-bearing ciliate, somewhat masked by debris, shown in (**E**; epifluorescence of DAPI staining at slightly higher magnification). Note nucleus. **(F)** Ciliate from 607 c10 shown in double exposure of DIC and DAPI epifluorescence. Note macro- and micronucleus. Scales: **B** = 50 μm; **C** = 10 μm; **D**, **E** = 25 μm; **F** = 10 μm.

**FIGURE 6:**
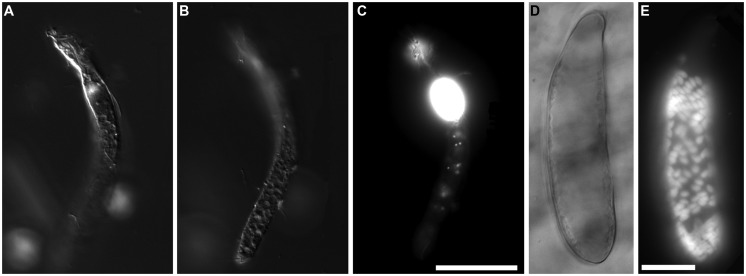
**Light micrographs of protists from Discovery DHAB. (A–C)** Ciliate from 609 c14 (mid-halocline). **(A–C)** Paired images showing DIC **(A,B)** and epifluorescence **(C)** of DAPI staining. Note the nucleus **(C)** and lack of abundant prokaryotic associates **(C)**. **(D,E)** Paired images showing DIC **(D)** and epifluorescence **(E)** of protist from 610 c9 (lower halocline). Note lack of obvious single large nucleus and presence of possible endobionts or parasites/scavengers. Scale bars = 20 μm.

**FIGURE 7:**
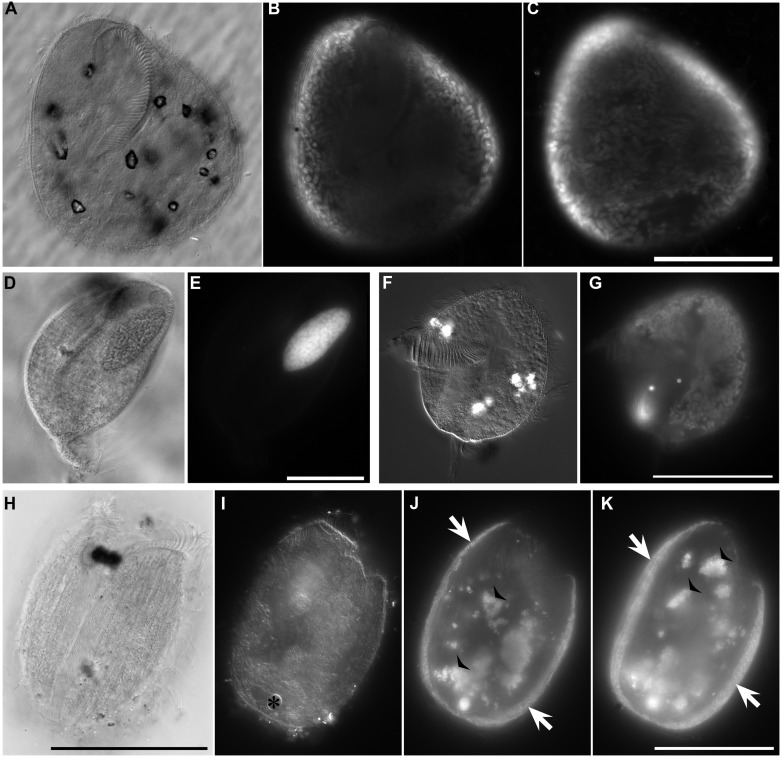
**Light micrographs of ciliates from L’ Atalante DHAB. (A,C)** Ciliate from 611 c5 (upper halocline); paired images showing DIC **(A)** and epifluorescence **(B,C)** of DAPI staining. Note the nucleus in **(A)**; DAPI staining of the nucleus also occurred (not shown). Also note the abundant endobionts **(B,C)**, mostly at protist periphery. **(D,E)** Specimen from 611 c10 (mid-halocline); paired images showing DIC **(D)** and epifluorescence **(E)** of DAPI staining. Note the nucleus and lack of prokaryotic associates. **(F,G)** Specimen from 611 c10; paired images showing DIC **(F)** and epifluorescence **(G)** of DAPI staining. Note the nucleus (somewhat out of the plane of view in **G**) and abundant endobiont associates. **(H–K)** Specimen from 611 c10; DIC image **(H)** along with paired images **(I–K)** showing darkfield **(I)** and epifluorescence **(J,K)** of DAPI staining. Note the nucleus (* in **I**), ectobionts (arrows) and aggregated endobiont associates (arrowheads). Scales: **C,G** = 100 μm; **E** = 25 μm; **H,K** = 50 μm.

**FIGURE 8:**
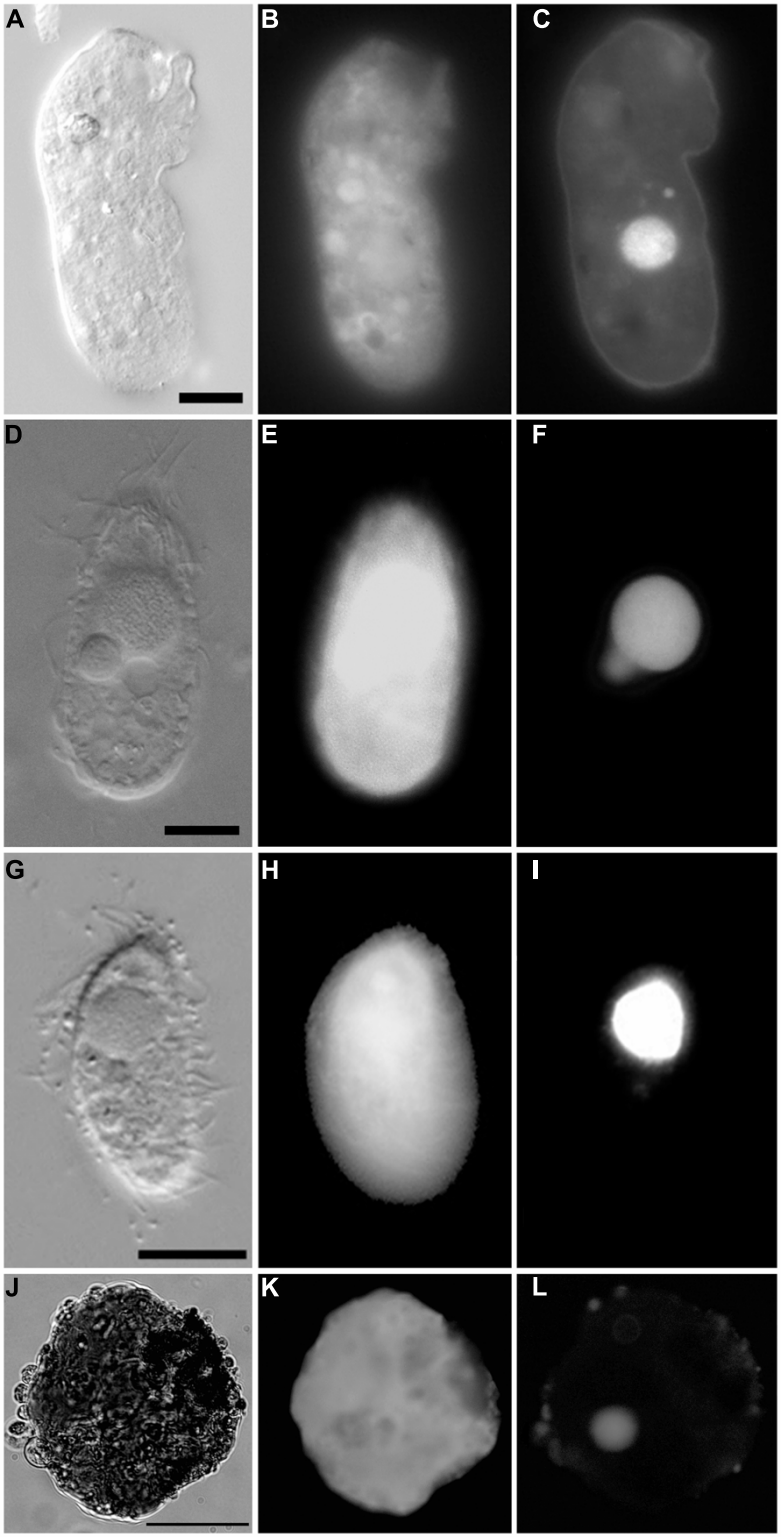
**Light micrographs of protists incubated in both CellTracker Green and DAPI. (A–C)** Specimen from 607-608 cB (Urania control); paired images showing DIC **(A)** and epifluorescence **(B,C)** of CellTracker Green labeling **(B)** and DAPI **(C)** staining. Note the fluorescence of the cytoplasm, the nucleus **(C)**, and absence of abundant associated prokaryotes. **(D–F)** Ciliate from 607-608 cE (Urania halocline); paired images showing DIC **(D)** and epifluorescence **(E,F)** of CellTracker Green labeling **(E)** and DAPI **(F)** staining. Note fluorescence of the cytoplasm, the macronucleus, and micronucleus **(F)** and lack of prokaryotic associates. **(G–I)** Ciliate from 609-610 cH (Discovery upper halocline); paired images showing DIC **(G)** and epifluorescence **(H,I)** of CellTracker Green labeling **(H)** and DAPI **(I)** staining. Note the lack of prokaryotic associates. **(J–L)** Unidentified protist from 609-610 cL (Discovery lower halocline). Note the nucleus and scattered bacteria on exterior. Scales: **A,G** = 10 μm, **D** = 100 μm, **J** = 50 μm.

Protist morphotypes were detected from every habitat (normoxic control, upper halocline, mid-halocline, lower halocline) in each sample examined for this purpose (**Table 2**). A number of morphotypes were observed (**Figures 4**–**8**). For example, what appeared to be a thecate foraminifera was observed in a control sample (**Figure 4A**), but none of these were noted in halocline samples. Small (3–5 micron) eukaryotes that were seen under fluorescence microscopy appeared to be consistent with flagellates, dominated microscopic counts in terms of abundance, but the most photogenic protists were ciliates (**Figures 5–7**). Not all protists had discernable nuclei. When no nuclei or few prokaryotes via DAPI were observed, the specimen was deemed dead (**Figures 4C,D**). In some cases, nuclei were visible in both DIC and DAPI images (e.g., **Figures 5A,B,D,E** and **7A,C,D,E** but DAPI staining of nucleus not shown). In other cases, even though prokaryotic associates (see below) were discernable by DAPI staining, nuclei that should also be discernable via DAPI staining were not obvious (e.g., **Figures 6D,E**). Some samples (i.e., 610 c14; 610 cL; 611 c10; 611 cC) had numerous filamentous bodies that were likely fungal hyphae or, perhaps, filamentous bacteria similar to *Beggiatoa*, although well-developed *Beggiatoa* mats were not observed. Moreover, *Beggiatoa* sequences were not detected in our samples (Kormas et al., unpublished data). Metazoa that were found (Bernhard et al., unpublished data) are not considered in this contribution.

### PROKARYOTE ASSOCIATES

Although protist populations were not quantified for presence of prokaryotic associates (i.e., endobionts, ectobionts), many ciliates had endobionts (**Figures 5B,C,E** and **7B,C,G,J,K**), while others did not (e.g., **Figures 5C,F**, **7E**, and **8F**). Ectobionts were noted on a few specimens (**Figures 7J,K**), and clumps of endobionts were seen in a halocline ciliate from L’ Atalante (**Figures 7J,K**).

### PROTIST VIABILITY SUGGESTED BY CELLTRACKER GREEN

In the aliquots examined for both CellTracker Green labeling and DAPI labeling, esterase-positive (i.e., CellTracker Green labeled) protists were observed that had easily distinguishable nuclei, either in addition to or exclusive from any associated ecto- and/or endobionts (**Figure 8**). Such specimens were observed in normoxic and some halocline samples (i.e., 607–608 cB and cE; 609–610 cL).

### MICROSCOPIC QUANTIFICATION OF PROTISTS

**FIGURE 9:**
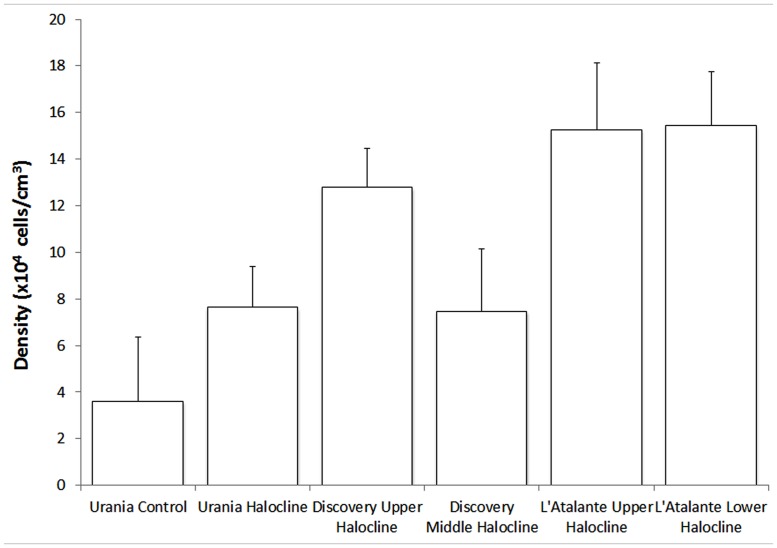
**Number of protists per unit cubic centimeter of sediment (*in situ*), presented for each habitat.** Error bars represent the SD resultant from counts of triplicate subsamples from each sample. Urania control = 607-08 cB; Urania halocline = 607-08 cE; Discovery upper halocline = 610 c14; Discovery mid-halocline = 609-10 cL; L’ Atalante upper halocline = 611 c10; L’ Atalante lower halocline = 611 c17.

Densities of protists varied within haloclines at Discovery Basin and between basins (**Figure 9**). The mean protist density of the five halocline samples (mean ± SD; 11.71 × 10^4^ cells/cm^3^ ± 3.94) was significantly higher than the protist density at the normoxic (Urania control) sample, (3.60 × 10^4^ cells; one-sample *t*-test, *t* = 4.599; df = 4, *p* = 0.010; **Figure 9**).

### MOLECULAR SIGNATURES OF PROTISTS AND FUNGI

After quality trimming and removal of non-protistan sequences and fungal sequences, the number of eukaryotic reads obtained from each sample analyzed for eukaryote diversity varied drastically, with the most reads (6546) in the Discovery upper halocline, followed by both L’ Atalante halocline samples (539 reads in lower halocline, 390 reads in upper halocline), and Urania halocline (82 reads) and Urania normoxic control (75 reads). For two of the samples analyzed, the number of curated reads was too low and these were excluded from the graphical representation. Discovery mid-halocline had a total of 8 reads, where most were Ustilaginomycotina Basidiomycota (not shown). Discovery lower halocline had a total of 6 reads, where most were Chlorophyceae (not shown).

**FIGURE 10:**
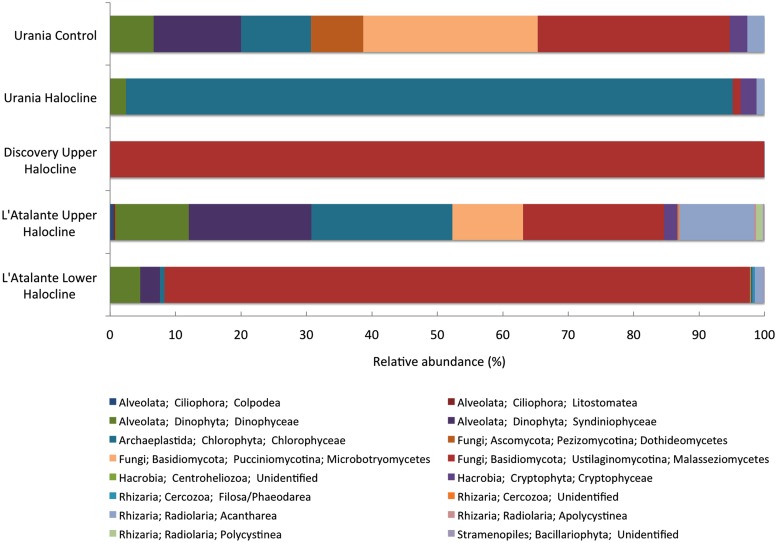
**Relative abundance (%) of rRNA signatures of unicellular eukaryotic groups of the DHAB sediments based on V4 SSU rRNA pyrotags clustered at 97% similarity.** Taxonomic assignments were performed using BLAST implemented in QIIME against SILVA 111 database.

A higher number of orders of eukaryotic taxa based on SSU rRNA signatures was detected in the Urania aerated control sample compared to the halocline samples, implying that alpha diversity may be lower inside these DHABs. The only exception was L’ Atalante upper halocline, which also had relatively high diversity (**Figure 10**). In four of these five samples, the majority of eukaryotic reads were fungal, with most being affiliated to Ustilaginomycotina Basidiomycota. L’ Atalante upper halocline was dominated by Chlorophyceae. Other groups with high diversity were Syndiniophyceae (L’ Atalante upper halocline, Urania aerated) and Dinophyceae (L’ Atalante upper halocline) Dinophyta. Rhizarians were also present, sometimes in considerable proportions (13%, L’ Atalante upper halocline), mostly as Radiolaria.

## DISCUSSION

Based on microscopic and sequencing analyses, protists and fungi were present in all our sediment samples, including lower halocline (higher salinity) samples. Eukaryote densities in the control (normoxic) sediments were roughly similar to densities of other protist communities in some oxygenated bathyal sediments (i.e., off Southern California, CA, USA; Bernhard et al., 2000), but higher than those in other normoxic bathyal sediments (i.e., Coral Sea; Alongi, 1987). Densities of protists in the DHAB halocline sediments were approximately equal to those in bathyal chemocline sediments of a silled basin off Baja California (i.e., Soledad Basin; Bernhard and Buck, 2004) but were only about one third those of the eukaryote community of bacterial mat chemocline sediments of Santa Barbara Basin, CA, USA (Bernhard et al., 2000). The detected densities might be considered surprisingly high given the oligotrophic nature of the Mediterranean (e.g., Kress et al., 2003; Pujo-Pay et al., 2011; Huertas et al., 2012; Tanhua et al., 2013). Even though none of the haloclines supported well-developed bacterial mats, our quantitative data suggests that the haloclines of these DHABs are inhabited by diverse eukaryotes, seemingly supporting higher numbers of eukaryotic microbes than the surrounding normoxic deposits. In deep oxic and oligotrophic Mediterranean waters above DHABs, protist counts are very low, ∼15-223 cells mL^-1^, but within the water-column haloclines (e.g., Urania halocline), protist numbers can rival those in near-surface waters, ∼1071 ± 117cells mL^-1^ (Pachiadaki et al., in press). Our quantitative observations from the three DHAB benthic halocline habitats had two orders of magnitude more protists than in documented DHAB water-column haloclines (Edgcomb et al., 2011d; Pachiadaki et al., in press).

**FIGURE 11:**
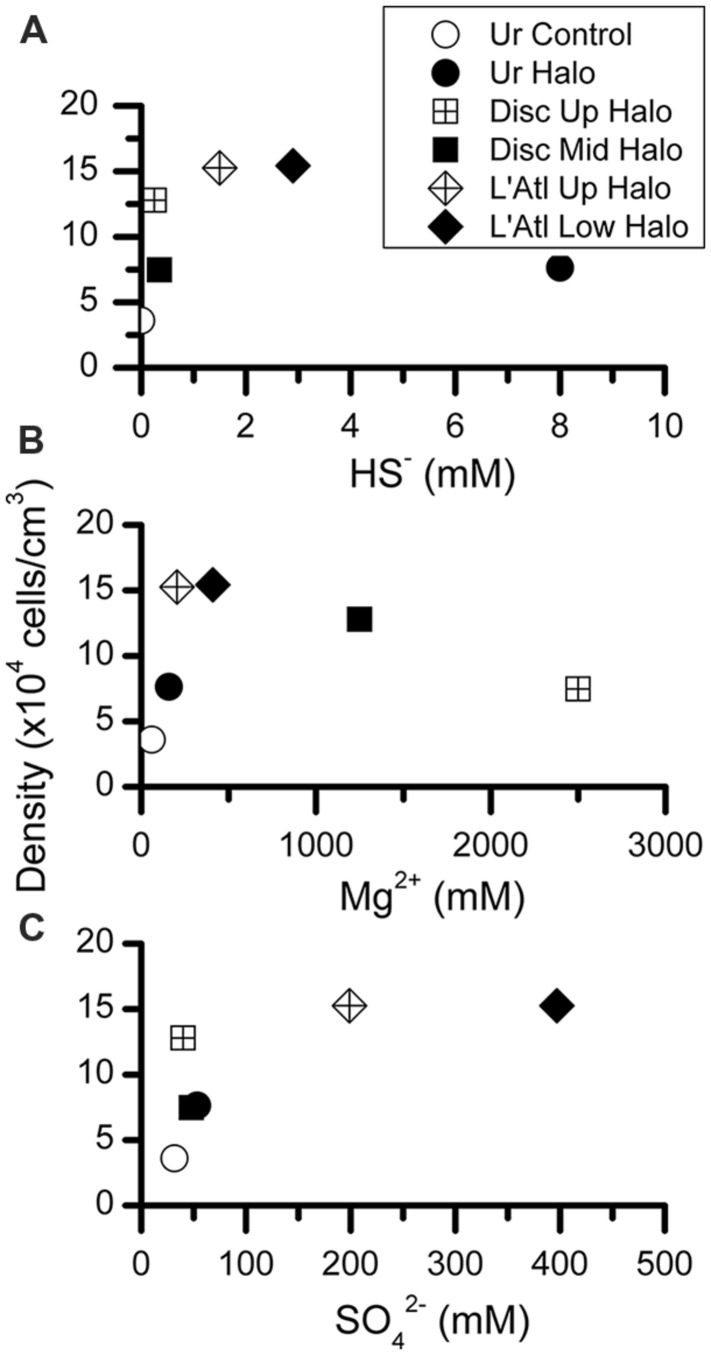
**Abundances of protists per cubic centimeter, calculated from microscopic counts, presented with respect to inferred chemical parameter in overlying waters.** Chemical constituent data from van der Wielen et al. (2005).

To determine whether protist density showed a relationship with salinity or chemical constituent, we plotted our protist density data (**Figure 9**) with published data on the seawater chemistry of each DHAB (**Table 1**). In general, higher protist densities occurred with higher concentrations of HS^-^, Mg^2+^, and SO_4_^2^ to a threshold (**Figure 11**). Protist densities decreased with the highest concentrations of sulfide and magnesium. No relationship was apparent between salinity and protist density. Refractometer readings of salinity for athalassohaline brines such as Discovery are problematic, and therefore comparisons to thalassohaline brines such as Urania and L’ Atalante are difficult. Protist density may relate to the differing cation chemistries in each of the DHABs (**Table 1**) and whether encountered specimens were living or dead (see below). Statistical analyses to test for trends were not performed as protist data from replicate cores at each habitat and halocline site were not available.

Although a minority, a considerable number of protists supported associated prokaryotes. These putative symbionts were more commonly endobionts than ectobionts. In one case, what appeared to be clumped endobionts were noted (**Figures 7J,K**). This pattern is somewhat similar to observations of another ciliate with multiple endobionts in a structured configuration, inhabiting chemocline sediments (Edgcomb et al., 2011c). Alternatively, these clumped endobionts may be in food vacuoles. It is possible that some of the DAPI signals detected in some eukaryotic specimens were actually of other smaller protists. For example **Figures 6D,E** may show numerous parasitic protists inside a remnant cell of another eukaryote of unknown affinity. We recovered some sequences of Syndiniales, which are parasitic dinoflagellates known to sometimes occur in deep-sea hydrothermal vent settings (e.g., Sauvadet et al., 2010).

Observed protists may not have been living individuals but merely locales of prokaryote infestation. Specifically, anaerobic bacteria and/or archaea may have inhabited protists as suggested by the lack of a nucleus/nuclei in some protists (**Figures 7D,E** and **8B,C**) yet presence of copious endo- or ectobionts evidenced by DAPI staining. Prokaryote presence within degrading foraminifera cytoplasm has been documented in hydrocarbon seep habitats (Bernhard et al., 2010b). Ribosomal RNA can be preserved in the high salinity environments of DHAB haloclines and brines (Hallsworth et al., 2007), and therefore, an unknown fraction of taxa whose rRNA signatures were detected here may represent inactive or dead and preserved cells. The detection of rRNA signatures of the photosynthetic Chlorophyceae in halocline samples in this study supports this suggestion, although the same signatures were detected in oxic normal saline sediments, which argues against preservation, and for the possibility that they may still be active. Ribosomal RNA signatures of additional photosynthetic organisms have previously been reported from deep marine sediments (Orsi et al., 2013). The potential for preservation of rRNA, particularly under anoxia and hypersaline conditions means that we must interpret rRNA-based data from these habitats cautiously, and find support for activity from microscopical observations and other types of molecular data. Furthermore, although here we report positively labeled eukaryotes with CellTracker Green (i.e., esterase activity), bacteria also react to this fluorogenic probe (Bernhard et al., 2003) could cause the eukaryotes to appear positively labeled. In sum, the protists present in the halocline samples may have been dead and only appeared to be inhabitants of these extreme habitats. Indeed, it may be that eukaryote carcasses, or “pickled protists” have been in the DHAB haloclines and brines since their formation. Estimates of Eastern Mediterranean DHAB formation are, at a minimum, 2000 years (Wallmann et al., 1997).

The presence of pickled protists may explain some of the observed trends of protist abundance (densities) with respect to water chemistry (**Figure 11**), at least for some constituents. More specifically, for Mg^2+^ and HS^-^, it is plausible that the observed protist peak abundances coincide with the approximate maximum tolerance of protists for the constituent being considered (**Figures 11A,C**). Regarding Mg^2+^, although an upper limit for prokaryotic life may be ∼2 M (Hallsworth et al., 2007), concentrations exceeding 0.2 M MgCl_2_ may be inhospitable to protists (**Figure 11A**). Similarly, DHAB environments exceeding ∼1.5 mM HS^-^ (**Figure 10C**) and ∼200 mM SO_4_^2-^ (**Figure 11B**) may not support living protist populations. Thresholds probably also depend on concentrations of additional constituents and their possible synergistic interactions in these brines, so generalizations involving habitats with typical marine salinities are likely invalid.

At least some protists were living in normoxic and halocline DHAB sediments, as evidenced by positive CellTracker Green labeling along with DAPI analysis indicating the lack of associated microbes but the presence of a nucleus or nuclei (e.g., **Figure 8**). Importantly, we do not assert positive DAPI staining indicates viability, as we are aware that DNA can be preserved in DHAB brines (e.g., Borin et al., 2008). Regardless, at least some protists lacking prokaryotic associates appear to inhabit upper, mid-, and lower DHAB halocline sediments.

Our assertion that filamentous items are most likely Fungi is supported by the recent microscopic observations documenting filamentous fungi in the oxycline and deep anoxic zones of the Black Sea (Sergeeva and Kopytina, 2014). The rRNA sequence data suggest that Fungi dominate the eukaryotic community in the MgCl_2_-dominated Discovery basin upper halocline, and in the L’Atlante basin lower halocline, confirming previous findings of high abundance of Fungi in the lower haloclines of DHAB water columns (Stock et al., 2012; Pachiadaki et al., 2014). Fungi were also reported to dominate the DNA libraries of the hypersaline sediments from a Red Sea brine pool (Wang et al., 2014). In that study, cultured representatives from those brine sediments were affiliated to Ascomycota, while in the present study the majority of retrieved ribotypes of Fungi from the halocline sediments were identified as Basidiomycota, suggesting that different halotolerant (or halophilic) fungal groups might have adapted to different local environments. The libraries from the lower haloclines of both Discovery and L’ Atalante share the same most abundant OTU. This OTU showed high nucleotide identity (99% with 100% sequence coverage) with an uncultured *Malassezia* isolate [KC487833] from the hypersaline Lake Tyrrell, VIC, Australia (Heidelberg et al., 2013). Recently and Amend (2014) provided evidence that the genus *Malassezia* is cosmopolitan and probably of great importance in deep-sea extreme environments. Interestingly, we have retrieved the same OTU (99% of sequence similarity) in the anoxic lower halocline water layer of another DHAB, Thetis (JF308281; Stock et al., 2012), as well as in the anoxic marine Cariaco Basin (GU824665; Edgcomb et al., 2011a), in the anoxic fjord Saanich Inlet (HQ866112; Orsi et al., 2012) and in the deep subsurface marine sediment of Peru Margin (GU972524; Edgcomb et al., 2011b). The marine species of *Malassezia* remain uncultured, so we cannot yet infer much about the ecology and trophic status of this OTU found in our survey.

Our data also support the notion that increasing salinity (e.g., L’ Atalante basin rRNA profiles) leads to a decrease in relative abundance of selected groups, e.g., a decrease in abundance within Dinophyceae, Cryptophyceae, Centroheliozoa, and Acantharea in the lower halocline sample from L’ Atalante basin relative to the upper halocline. The dominance of chlorophyte signatures in the Urania basin upper halocline sample suggests decreased protist diversity possibly due to the relatively high sulfide concentrations there.

The application of metatranscriptomics to these communities may be the only feasible means at present to fully resolve eukaryote activity (i.e., gene expression) and their inhabitation of these DHAB haloclines (Pachiadaki et al., unpublished data). Future efforts may reveal novel taxa and heretofore unknown biochemical pathways in these spatially restricted benthic habitats.

## AUTHOR CONTRIBUTIONS

Joan M. Bernhard and Virginia P. Edgcomb designed this study and drafted the manuscript. Joan M. Bernhard, Konstantinos Kormas, Maria G. Pachiadaki, and Colin Morrison collected the samples. All authors performed analyses and contributed to data analysis. All authors read and approved the final manuscript.

## Conflict of Interest Statement

The authors declare that the research was conducted in the absence of any commercial or financial relationships that could be construed as a potential conflict of interest.

## References

[B1] Alexander E. Stock A. Breiner H. W. Behnke A. Bunge J. Yakimov M. M. (2009). Microbial eukaryotes in the hypersaline anoxic L’ Atalante deep-sea basin. *Environ. Microbiol.* 11 360–381 10.1111/j.1462-2920.2008.01777.x 18826436

[B2] Alongi D. M. (1987). The distribution and composition of deep-sea microbenthos in a bathyal region of the Western Coral Sea. *Deep Sea Res. Part A Oceanogr. Res. Pap.* 34 1245–1254 10.1016/0198-0149(87)90074-4

[B3] Amend A. (2014). From dandruff to deep-sea vents: *Malassezia*-like Fungi are ecologically hyper-diverse. *PLoS Pathog.* 10:e1004277 10.1371/journal.ppat.1004277 PMC4140847 25144294

[B4] Bernhard J. M. (2003). Potential symbionts in bathyal foraminifera. *Science* 299 861–861 10.1126/science.1077314 12574621

[B5] Bernhard J. M. Barry J. P. Buck K. R. Starczak V. R. (2009). Impact of intentionally injected carbon dioxide hydrate on deep-sea benthic foraminiferal survival. *Global Change Biol.* 15 2078–2088 10.1111/j.1365-2486.2008.01822.x

[B6] Bernhard J. M. Bowser S. S. (2008). Peroxisome proliferation in foraminifera inhabiting the chemocline: an adaptation to reactive oxygen species exposure? *J. Eukaryot. Microbiol.* 55 135–144 10.1111/j.1550-7408.2008.00318.x 18460150 PMC2744378

[B7] Bernhard J. M. Buck K. R. (2004). “Eukaryotes of the cariaco, soledad, and santa barbara basins: protists and metazoans associated with deep-water marine sulfide-oxidizing microbial mats and their possible effects on the geologic record,” in *Sulfur Biogeochemistry – Past and Present*, eds Amend J. P. Edwards K. J. Lyons T. W. (Boulder: GSA Special Paper), 35–47.

[B8] Bernhard J. M. Buck K. R. Farmer M. A. Bowser S. S. (2000). The santa barbara basin is a symbiosis oasis. *Nature* 403 77–80 10.1038/47476 10638755

[B9] Bernhard J. M. Goldstein S. T. Bowser S. S. (2010a). An ectobiont-bearing foraminiferan, bolivina pacifica, that inhabits microxic pore waters: cell-biological and paleoceanographic insights. *Environ. Microbiol.* 12 2107–2119 10.1111/j.1462-2920.2009.02073.x 21966906

[B10] Bernhard J. M. Martin J. B. Rathburn A. E. (2010b). Combined Bernhard carbonate carbon isotopic and cellular ultrastructural studies of individual benthic foraminifera: 2. Toward an understanding of apparent disequilibrium in hydrocarbon seeps. *Paleoceanography* 25 10.1029/2010pa001930

[B11] Bernhard J. M. Visscher P. T. Bowser S. S. (2003). Submillimeter life positions of bacteria, protists, and metazoans in laminated sediments of the santa barbara basin. *Limnol. Oceanogr.* 48 813–828 10.4319/lo.2003.48.2.0813

[B12] Borin S. Crotti E. Mapelli F. Tamagnini I. Corselli C. Daffonchio D. (2008). DNA is preserved and maintains transforming potential after contact with brines of the deep anoxic hypersaline lakes of the Eastern Mediterranean Sea. *Saline Syst.* 4 10 10.1186/1746-1448-4-10 PMC2531117 18681968

[B13] Bragg L. Stone G. Imelfort M. Hugenholtz P. Tyson G. W. (2012). Fast, accurate error-correction of amplicon pyrosequences using Acacia. *Nat. Methods* 9 425–426 10.1038/nmeth.1990 22543370

[B14] Cai W. J. Sayles F. L. (1996). Oxygen penetration depths and fluxes in marine sediments. *Mar. Chem.* 52 123–131 10.1016/0304-4203(95)00081-x

[B15] Camerlenghi A. (1990). Anoxic basins of the Eastern Mediterranean: geological framework. *Mar. Chem.* 31 1–19 10.1016/0304-4203(90)90028-B

[B16] Caporaso J. G. Kuczynski J. Stombaugh J. Bittinger K. Bushman F. D. Costello E. K. (2010). QIIME allows analysis of high-throughput community sequencing data. *Nat. Methods* 7 335–336 10.1038/nmeth.f.303 20383131 PMC3156573

[B17] Danovaro R. Dell’anno A. Pusceddu A. Gambi C. Heiner I. Kristensen R. M. (2010). The first metazoa living in permanently anoxic conditions. *BMC Biol.* 8:30 10.1186/1741-7007-8-30 PMC2907586 20370908

[B18] Edgcomb V. Orsi W. Bunge J. Jeon S. Christen R. Leslin C. (2011a). Protistan microbial observatory in the Cariaco Basin, Caribbean. *I.* Pyrosequencing vs Sanger insights into species richness. *ISME J.* 5 1344–1356 10.1038/ismej.2011.6 21390079 PMC3146274

[B19] Edgcomb V. P. Beaudoin D. Gast R. Biddle J. F. Teske A. (2011b). Marine subsurface eukaryotes: the fungal majority. *Environ. Microbiol.* 13 172–183 10.1111/j.1462-2920.2010.02318.x 21199255

[B20] Edgcomb V. P. Leadbetter E. R. Bourland W. Beaudoin D. Bernhard J. M. (2011c). Structured multiple endosymbiosis of bacteria and archaea in a ciliate from marine sulfidic sediments: a survival mechanism in low oxygen, sulfidic sediments? *Front. Microbiol.* 2:55 10.3389/fmicb.2011.00055 PMC3153031 21833311

[B21] Edgcomb V. P. Orsi W. Breiner H. W. Stock A. Filker S. Yakimov M. M. (2011d). Novel active kinetoplastids associated with hypersaline anoxic basins in the Eastern Mediterranean deep-sea. *Deep Sea Res. Part I Oceanogr. Res. Pap.* 58 1040–1048 10.1016/j.dsr.2011.07.003

[B22] Edgcomb V. P. Orsi W. Leslin C. Epstein S. S. Bunge J. Jeon S. (2009). Protistan community patterns within the brine and halocline of deep hypersaline anoxic basins (DHABs) in the Eastern Mediterranean Sea. *Extremophiles* 13 151–167 10.1007/s00792-008-0206-2 19057844

[B23] Esteban G. Fenchel T. Finlay B. (1995). Diversity of free-living morphospecies in the ciliate genus Metopus. *Arch. Protist.* 146 137–164 10.1016/S0003-9365(11)80106-5

[B24] Fenchel T. Finlay B. (1995). *Ecology and Evolution in Anoxic Worlds.* Oxford: Oxford University Press.

[B25] Ferrer M. Golyshina O. V. Chernikova T. N. Khachane A. N. Dos Santos V. Yakimov M. M. (2005). Microbial enzymes mined from the Urania deep-sea hypersaline anoxic basin. *Chem. Biol.* 12 895–904 10.1016/j.chembiol.2005.05.020 16125101

[B26] Ferrer M. Werner J. Chernikova T. N. Bargiela R. Fernandez L. La Cono V. (2012). Unveiling microbial life in the new deep-sea hypersaline Lake Thetis. Part II: a metagenomic study. *Environ. Microbiol.* 14 268–281 10.1111/j.1462-2920.2011.02634.x 22040283

[B27] Hallsworth J. E. Yakimov M. M. Golyshin P. N. Gillion J. L. M. D’auria G. Alves F. D. L. (2007). Limits of life in MgCl2-containing environments: chaotropicity defines the window. *Environ. Microbiol.* 9 801–813 10.1111/j.1462-2920.2006.01212.x 17298378

[B28] Heidelberg K. B. Nelson W. C. Holm J. B. Eisenkolb N. Andrade K. Emerson J. B. (2013). Characterization of eukaryotic microbial diversity in hypersaline Lake Tyrrell, Australia. *Front. Microbiol.* 4:115 10.3389/fmicb.2013.00115 PMC3651956 23717306

[B29] Huertas I. E. Rios A. F. Garcia-Lafuente J. Navarro G. Makaoui A. Sanchez-Roman A. (2012). Atlantic forcing of the Mediterranean oligotrophy. *Global Biogeochem. Cycles* 26:GB2022 10.1029/2011gb004167

[B30] Kress N. Manca B. B. Klein B. Deponte D. (2003). Continuing influence of the changed thermohaline circulation in the eastern Mediterranean on the distribution of dissolved oxygen and nutrients: physical and chemical characterization of the water masses. *J. Geophys. Res. Oceans* 108:8109 10.1029/2002jc001397

[B31] Orcutt B. Boetius A. Elvert M. Samarkin V. Joye S. B. (2005). Molecular biogeochemistry of sulfate reduction, methanogenesis and the anaerobic oxidation of methane at gulf of Mexico cold seeps. *Geochim. Cosmochim. Acta* 69 4267–4281 10.1016/j.gca.2005.04.012

[B32] Orsi W. Biddle J. Edgcomb V. P. (2013). Deep sequencing of subseafloor eukaryotic rRNA reveals active fungi across marine subsurface provinces. *PLoS ONE* 8:e56335 10.1371/journal.pone.0056335 PMC3572030 23418556

[B33] Orsi W. Song Y. C. Hallam S. Edgcomb V. (2012). Effect of oxygen minimum zone formation on communities of marine protists. *ISME J.* 6 1586–1601 10.1038/ismej.2012.7 22402396 PMC3400406

[B34] Pachiadaki M. Taylor C. Oikomomou A. Yakimov M. M. Stoeck T. Edgcomb V. P. (in press) Grazing studies conducted in situ reveal protist turnover of bacterial biomass in the deep E. Mediterranean Sea. *Deep Sea Res.* II.

[B35] Pachiadaki M. Yakimov M. M. Lacono V. Leadbetter E. R. Edgcomb V. (2014). Unveiling microbial activities along the halocline of thetis, a deep-sea hypersaline anoxic basin. *ISME J.* 10.1038/ismej.2014.100 PMC4260694 24950109

[B36] Pujo-Pay M. Conan P. Oriol L. Cornet-Barthaux V. Falco C. Ghiglione J. F. (2011). Integrated survey of elemental stoichiometry (C, N, P) from the western to eastern Mediterranean Sea. *Biogeosciences* 8 883–899 10.5194/bg-8-883-2011

[B37] Reimers C. E. Ruttenberg K. C. Canfield D. E. Christiansen M. B. Martin J. B. (1996). Porewater pH and authigenic phases formed in the uppermost sediments of the Santa Barbara Basin. *Geochim. Cosmochim. Acta* 60 4037–4057 10.1016/S0016-7037(96)00231-1

[B38] Sauvadet A. L. Gobet A. Guillou L. (2010). Comparative analysis between protist communities from the deep-sea pelagic ecosystem and specific deep hydrothermal habitats. *Environ. Microbiol.* 12 2946–2964 10.1111/j.1462-2920.2010.02272.x 20561018

[B39] Sergeeva N. G. Kopytina N. I. (2014). The first marine filamentous Fungi discovered in the bottom sediments of the oxic/anoxic interface and in the bathyal zone of the Black Sea. *Turkish J. Fish. Aquat. Sci.* 14 497–505 10.4194/1303-2712-v14-2-21

[B40] Starink M. Bargilissen M. J. Bak R. P. M. Cappenberg T. E. (1994). Quantitative centrifugation to extract benthic protozoa from fresh-water sediments. *Appl. Environ. Microbiol.* 60 167–173. 16349148 10.1128/aem.60.1.167-173.1994 PMC201285

[B41] Stock A. Breiner H. W. Pachiadaki M. Edgcomb V. Filker S. La Cono V. (2012). Microbial eukaryote life in the new hypersaline deep-sea basin Thetis. *Extremophiles* 16 21–34 10.1007/s00792-011-0401-404 22009262

[B42] Stock A. Edgcomb V. Orsi W. Filker S. Breiner H. W. Yakimov M. M. (2013a). Evidence for isolated evolution of deep-sea ciliate communities through geological separation and environmental selection. *BMC Microbiol.* 13:150 10.1186/1471-2180-13-150 PMC3707832 23834625

[B43] Stock A. Filker S. Yakimov M. M. Stoeck T. (2013b). “Deep hypersaline anoxic basins as model systems for environmental selection of microbial plankton,” in *Polyextremophiles, Life Under Multiple forms of Stress* eds Seckbach J. Oren A. Stan-Lotter H. (Dordrecht: Springer), 501–515.

[B44] Tanhua T. Hainbucher D. Schroeder K. Cardin V. Alvarez M. Civitarese G. (2013). The Mediterranean Sea system: a review and an introduction to the special issue. *Ocean Sci.* 9 789–803 10.5194/os-9-789-2013

[B45] van der Wielen P. Bolhuis H. Borin S. Daffonchio D. Corselli C. Giuliano L. (2005). The enigma of prokaryotic life in deep hypersaline anoxic basins. *Science* 307 121–123 10.1126/science.1103569 15637281

[B46] van der Wielen P. Heijs S. K. (2007). Sulfate-reducing prokaryotic communities in two deep hypersaline anoxic basins in the Eastern Mediterranean deep sea. *Environ. Microbiol.* 9 1335–1340 10.1111/j.1462-2920.2006.01210.x 17472645

[B47] Visscher P. T. Beukema J. Van Gemerden H. (1991). In situ characterization of sediments- measurments of oxygen and sulfide profiles with a novel combined needle electrode. *Limnol. Oceanogr.* 36 1476–1480 10.4319/lo.1991.36.7.1476

[B48] Wallmann K. Suess E. Westbrook G. H. Winckler G. Cita M. B. (1997). Salty brines on the Mediterranean sea floor. *Nature* 387 31–32 10.1038/387031a0

[B49] Wang Y. Zhang W. P. Cao H. L. Shek C. S. Tian R. M. Wong Y. H. (2014). Diversity and distribution of eukaryotic microbes in and around a brine pool adjacent to the Thuwal cold seeps in the Red Sea. *Front. Microbiol.* 5:37 10.3389/fmicb.2014.00037 PMC3922051 24575081

[B50] Yakimov M. M. La Cono V. Denaro R. D’Auria G. Decembrini F. Timmis K. N. (2007). Primary producing prokaryotic communities of brine, interface and seawater above the halocline of deep anoxic lake L’ Atalante, Eastern Mediterranean Sea. *ISME J.* 1 743–755 10.1038/ismej.2007.83 18059497

[B51] Yucel M. (2013). Down the thermodynamic ladder: a comparative study of marine redox gradients across diverse sedimentary environments. *Estuar. Coast. Shelf Sci.* 31 83–92 10.1016/j.ecss2013.07.013

